# A case study of health sector reform in Kosovo

**DOI:** 10.1186/1752-1505-4-7

**Published:** 2010-04-16

**Authors:** Valerie Percival, Egbert Sondorp

**Affiliations:** 1 Norman Paterson School of International Affairs, Carleton University, 1125 Colonel By Drive, Ottawa, ON, K1S 5B6, Canada; 2 London School of Hygiene and Tropical Medicine, Keppel St, London, WC1E 7HT, UK

## Abstract

The impact of conflict on population health and health infrastructure has been well documented; however the efforts of the international community to rebuild health systems in post-conflict periods have not been systematically examined. Based on a review of relevant literature, this paper develops a framework for analyzing health reform in post-conflict settings, and applies this framework to the case study of health system reform in post-conflict Kosovo. The paper examines two questions: first, the selection of health reform measures; and second, the outcome of the reform process. It measures the success of reforms by the extent to which reform achieved its objectives. Through an examination of primary documents and interviews with key stakeholders, the paper demonstrates that the external nature of the reform process, the compressed time period for reform, and weak state capacity undermined the ability of the success of the reform program.

## Introduction

This paper examines the efforts to rebuild the health system in Kosovo after the United Nations established administrative control of the province in 1999. In many ways, Kosovo represented the beginning of a new form of international engagement in countries emerging from armed conflict. The international community assumed administrative control of the province, including control over the health sector. However, unlike other post-conflict states such as Afghanistan and Iraq, the implementing environment in Kosovo was favourable: high levels of donor assistance were dispersed, the majority of the population supported the military intervention, and the province had reasonably high levels of human capital concentrated in a small geographic area situated in Europe. Because of these factors, Kosovo is an optimal case study to examine the efficacy of international engagement in post-conflict societies, including health reform.

Health reform is "sustained, purposive change to improve the efficiency, equity, and effectiveness of the health sector with the goal of improving health status, obtaining greater equity, and obtaining greater cost-effectiveness for services provided" [1]. In the analysis of health reform in Kosovo, the paper addresses two key questions:

• ***Policy Choices***: What health policies and programmes were selected as part of the health reform effort? Why were these policies selected?

• ***Policy Outcomes***: What factors impacted on the implementation of the health reform effort? What were the key successes and failures? Did health reform achieve its objectives?

The Kosovo health reform program was initially lauded as a success given the evidence-based, organized, and orderly nature of the policy generation process [2]. However, the implementation of these reforms was more problematic than their creation, and the outcome of reform has not met its promise. The case study is of interest to policy makers considering reforming health systems in post-conflict or crisis-affected states. While more comparative case studies are necessary before concrete policy recommendations can be developed, the Kosovo case provides a warning about the complex and difficult process of transforming and strengthening health systems.

## Methods

As no framework existed to guide the analysis of health reform in post-conflict settings, the paper first undertook a literature review to develop this framework. The authors searched the following sets of literature: the impact of conflict on health, health reform in Eastern Europe, and post-conflict reconstruction and peacebuilding efforts. The literature review produced a framework that identifies how the international engagement in the health sector interacts with the post-conflict social and political context.

The literature review also generated the following hypotheses on the factors that influenced the outcomes of reform.

*H1: External actors drove the health reform process: the policies selected reflected the objectives of the international community*.

*H2: Donors believed that reform could be achieved in a compressed time period, and gave more priority to the design than the implementation of reforms*.

*H3: State capacity in the post-conflict period is low, and external actors do not recognize the importance of state capacity in health reform*.

The externally driven nature of the reform process, the compressed nature of the time period for reform, and low state capacity undermined the ability of the health reform program to achieve its objectives.

The health reform process in Kosovo was analyzed through primary documents and interviews with 26 stakeholders active in the health sector. Local stakeholders were chosen based on their familiarity with the health reform process--most occupied positions within the Kosovo health system. While the majority of stakeholders were from Kosovo's capital city of Pristina, stakeholders from two of Kosovo's municipalities were also interviewed to integrate regional perspectives. The interviews were designed to evaluate the reform process.

The case study focuses on the initial five year period following reform (1999-2004) but also discusses the state of the Kosovo health sector today. This research confirms these hypotheses by demonstrating that the reform agenda was externally driven; the reform timetable was compressed - the international community was attempting 'too much, too fast,' and government capacity was low in the post-war social and political context. In addition to confirming these hypotheses, the research also found that the extreme politicization of the health sector impeded reform progress.

## Framework for Reform

**Figure 1:**
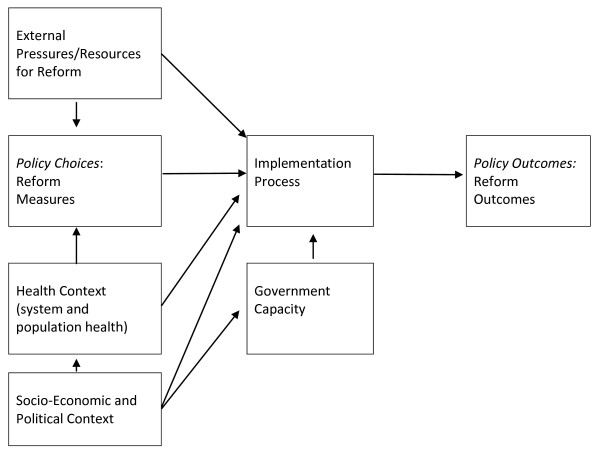
**Analysing Health Reform in Post-Conflict Settings**.

Post-conflict health reform remains an under-researched area, and as such, there are no pre-existing frameworks that analyze how health interventions interact with the post-conflict social, political and economic environment. The framework for examining health reform established in this paper outlines the process through which health reforms are developed and implemented in post-conflict settings and establishes the factors that are most important in shaping the outcomes of reform - the ability of reform to meet its objectives. This framework is presented in Figure 1, and its components are described below.

Pressure to undertake health reform arises from problems within the health care system such as high costs, poor performance, and poor infrastructure; as well as concerns regarding health status. Reform measures are composed of interventions that focus on the organisation of the system, health financing, and the structure of payments to health care providers and institutions. The objective of reform is to improve population health, improve health system performance (cost effectiveness), enhance risk protection, and heighten public satisfaction [3].

But the implementation of reform is always more challenging than the design. The experience of health reform in Eastern Europe (which had a similar health system design as Kosovo and a similar reform program) points to factors internal to the process of implementing reforms that derailed the reform effort: short time horizons for implementation, poor policy planning, financing reforms that failed due to weak administration capacity, the lack of enthusiasm for the reform program, and the difficulty to implement organisational change. The Eastern European reform program also points to factors external to health reforms, namely economic instability, unhealthy lifestyles, the lack of government capacity to implement reforms, and political instability all impacting on reform. The health reform experience in Eastern Europe also points to the important influence of multilateral organizations and donor governments in shaping the reform program [4,5].

Health reform is one component of a larger international intervention in post-conflict societies, and needs to be viewed as part of this larger wave of reform efforts. Significant pressure exists for donors and international agencies to use this opportunity to improve institutions, rather than simply refurbish the old ones [6]. The involvement of the international community brings a tremendous influx of donor resources that necessitates careful coordination of donor and non-governmental organization activity. Due to the influx of resources and multiple actors, evidence from previous international engagements suggests that a blueprint outlining the parameters of a future health system increases the sustainability of health interventions. Within those blueprints, specific health interventions are favoured by international actors, particularly the movement towards a primary care based system.

Moreover, the post-conflict environment is characterized by highly divisive politics, a weak economy, and low government capacity, all of which impact on the implementation of reform efforts. This context creates a difficult implementing environment.

To summarize the framework presented in Figure 1, post-conflict reform programs are launched as a result of poor population health and the need for rehabilitation of health infrastructure, often as a result of conflict-affected damage. External pressures for specific reform measures shape the selection of health interventions: the health reform process forms a component of the international community's effort to rebuild the state, and particular health reform measures are favoured by international actors. Short donor time horizons coupled with an ambitious reform agenda lead to compressed time frames for reform. Socio-economic and political forces undermine the capacity of the state to oversee and implement reform measures. These factors impact on the outcomes of reform, measured by the ability of the reform program to achieve its objectives. While reforms are launched to improve health status, the quality of health services, equitable access to those services, and the cost-effectiveness of the health system, evaluating health reform on these outcomes presents challenges. Health status and performance indicators can be difficult to examine due to the absence of health and management information systems. Moreover, the time lag between reform of the health system and improved health outcomes can be significant - particularly in a setting like Kosovo where chronic, rather than infectious diseases dominate.

## Background: Kosovo Health System

The health system in Kosovo, as elsewhere in Eastern Europe, was largely based on the Semashko model of healthcare delivery. The Semashko system of health care was utilized throughout the Soviet Union and Eastern Europe. It centralized decision-making and emphasized specialization of services. Polyclinics, located in major towns and municipalities, were the first point of contact for patients. General practitioners, dentists, paediatricians, and gynaecologists all practised at these clinics, and physiotherapy and basic diagnostic services were also available. The central government functioned as the purchaser as well as the provider of health care services. Yugoslavia adapted the Semashko model to reflect its version of socialism--a system of self-management. While favouring the delivery of health care by specialists, decision-making for the system was decentralized to hospitals and health centres. The healthcare system succeeded in expanding the provision of healthcare, and Kosovo saw dramatic health improvements: the mortality rate declined from 46 per 1,000 in 1956 to 29 per 1,000 in 1990 [7].

Under the 1974 Yugoslav Constitution, Kosovo had been granted autonomous status within the Republic of Serbia. This status was revoked by Belgrade in March 1989, initiating a decade of tension and conflict. The health sector became a natural battleground for the conflict between Kosovo's majority Albanian population and the federal government in Belgrade. The Belgrade Ministry of Health assumed control of the Kosovo health system, and directors and boards of health institutions were forced to report directly to Belgrade. Pristina University's medical faculty was closed, and the medical training of many students was interrupted. Sixty-four percent of ethnic Albanian health workers (an estimated 2,400 people) left their jobs: some were fired, others were subject to smear campaigns, while others left of their own accord. Four hundred and forty of those dismissed were specialist physicians. The gynaecology and maternity clinics were particularly hard hit, with all Albanian doctors working in these units leaving their positions. Those healthcare workers that remained in the system were required to speak Serbian and to write in Cyrillic [7].

Access to healthcare for Albanians suffered. Many Albanians lost their jobs after 1989, and as a result, lost their insurance coverage. During the 1990s, more than 50 percent of Albanians lacked a social insurance card needed to access the public health system.

To respond to this need, Albanians organized a parallel primary healthcare system in conjunction with the parallel government that was established in the early 1990s. This system, known as the Mother Theresa Society, operated 96 clinics throughout Kosovo, many in remote areas. Healthcare workers volunteered their services, with financing for supplies and medicines provided by a parallel tax system. Many Albanian health professionals also established private healthcare facilities, including clinics and laboratories, during this period.

Because Albanians were no longer able to receive medical training in their own language at Pristina University, they also created a parallel system of medical education. In the 1990s, 600 doctors and 1,200 nurses graduated from this parallel system. While this system provided students with a high degree of theoretical knowledge, clinical training was problematic given the lack of access of medical students to healthcare facilities. This left a generation of Albanian medical personnel with uncertain expertise and unrecognised qualifications.

Despite these efforts, population health deteriorated in the 1990s. The incidence rate of infectious diseases rose, immunisation rates declined, and vaccination coverage for children against polio, diphtheria, tetanus, pertussis, measles, mumps, and rubella fell below 60 percent, with some areas falling below 30 percent coverage. Polio re-emerged, with 52 cases reported between 1990 and 1997 [7].

Armed conflict broke out in 1998 between the Kosovo Liberation Army (KLA) and the Yugoslav Army and police. This conflict caused massive population displacements in rural areas of Kosovo. In the fall of 1998, UNHCR estimated that 200,000 Albanians were displaced. While many civilians fled to neighbouring Albania and Macedonia, others left their villages and took refuge in the hills of Kosovo. Health surveys showed that displacement, as well as the violence against Albanian civilians, took a devastating toll on population health. Between February 1998--roughly when the conflict between the KLA and Yugoslav authorities began--and June 1999, when NATO forces entered Kosovo, the crude mortality rate was 2.3 times higher than the pre-conflict baseline. War-related trauma was the major cause of death, with an estimated 12,000 deaths directly related to the war. The second leading cause of mortality was chronic disease [8].

In 1999, NATO undertook a military intervention in Kosovo. After two and a half months of aerial bombardments, the Yugoslav government agreed to the deployment of NATO troops in Kosovo and to the United Nations administering the province. On June 10, 1999, the United Nations Security Council passed Resolution 1244, which provided the legal foundation for United Nations control over the province. The United Nations Interim Administrative Mission in Kosovo (UNMIK) was formed, charged with building autonomous institutions of self-government. The mandate of UNMIK was to administer the province, while establishing and overseeing the development of provisional self-governing institutions. The NATO-led KFOR (the Kosovo Force) provided security. The international community was given sweeping powers to build autonomous self-government and undertake political, social, and economic reform.

## Applying the Framework to Kosovo

Above, the paper presented a framework for analyzing health care reform in a post-conflict setting. Below, we apply that framework to Kosovo, beginning with an overview of how the health context - both health infrastructure and population health problems, combined with external pressure for health reform to shape the selection of health reform measures. We then overview the health reform program, and outline the progress made toward implementing those reform measures. In applying this framework to Kosovo, the paper outlines how the post-conflict political context and weak government capacity combined to undermine progress on health reform.

### Health Context: The Health System

After the war, the parallel Mother Theresa Network was virtually abandoned. Albanian health professionals moved back into state health facilities, while most Serbian health professionals fled Kosovo - a result of the wave of violence directed against Serbs in the post-conflict period. In June 1999 the majority of the staff and patients at Pristina Hospital were Serb; by August 1999 the hospital staff and patients were almost exclusively Albanian.

The health system had been seriously weakened by the years of political and economic turmoil and by several months of conflict. Over 90 percent of the clinics of the parallel Mother Theresa Network were damaged or destroyed during the war, and many private clinics of Albanian health professionals had also been damaged. While public-health facilities were spared war-related damage, as Serbian doctors had staffed these clinics, the vast majority of them had been looted of supplies and equipment, and the infrastructure reflected years of neglect. The general collapse of public-service infrastructure--particularly water and electricity--deeply affected the health sector. Many hospitals lacked running water 24 hours a day.

Health clinics in rural areas suffered from an acute lack of personnel and equipment. Access to emergency and after-hours care was variable; while these services were often accessible in large cities, they were not available in rural areas. The availability of services through private practice had increased dramatically-while most Albanian health workers returned to public-health institutions, those that had developed private practices during the 1990s maintained them.

The quality of the public healthcare system was compromised by several factors. Access to primary care was inconsistent across regions and socioeconomic groups. Shortages of health personnel in rural areas, the specialised nature of healthcare in Kosovo, and the lack of a functioning referral system undermined the quality of care. Moreover the efficiency of services was minimal. Hospitals were composed of several separate buildings that contained separate clinics with their own laboratories, intensive-care facilities, and operating theatres. Services among the buildings were not shared, which resulted in duplication and inefficiency.

Kosovo also faced a shortage of physicians. The number of doctors was less than 2,500--on average 13 doctors for every 10,000 inhabitants (the European average is about 35 doctors per 10,000 inhabitants). Many doctors had trained in the parallel system and required skills upgrading. The exodus of Serb doctors in 1999 exacerbated this shortage. The number of doctors willing to work in rural areas was minimal, and rural residents often had to travel long distances to receive treatment.

While the shortage of physicians and the poor state of health facilities contributed to variable access to healthcare, economic factors also impacted on the ability of individuals to access health services. The World Bank found that the main barrier to healthcare was cost--despite the fact that healthcare was supposed to be free. Twenty-eight percent of those surveyed reported that they could not access health services due to expense. Over 95 percent of Albanians reported buying healthcare services, paying approximately three Euros for general expenses and five Euros in 'gifts' to healthcare providers. The average household spent 35 Euros annually on drugs [9].

The healthcare system was funded by revenue out of the Kosovo Consolidated Budget. This budget was a combination of donor funds and locally collected revenue. In the summer of 2000, the Department of Health instituted a co-payment system to fill a financing gap and support the primary care system (a financial penalty was incurred if patients bypassed the primary care system). These funding sources were inadequate, unsustainable, and slightly regressive. Donor contributions were waning, and both co-payments and under-the-table payments placed a heavy burden on the poor.

### Health Context: Population Health Status

With no reliable census in decades, Kosovo suffered from a lack of basic demographic data. Surveys indicated a young population with a mean age of 24.6 years. Twenty-three percent of the population was thought to be under 14, while 52 percent was between the ages of 15 and 49. The overall population balance appeared skewed: 50.3 percent of the population was male and 49.7 percent female, with a ratio of newborn male babies to females of 106:100 [10]. Women of childbearing age (between the ages of 15 to 45) constituted 56 percent of the female population and 26.2 percent of the total population [11].

The validity and reliability of health data was problematic, and the epidemiological situation was uncertain in 1999 and 2000. Hospital mortality studies showed that 12 percent of deaths were from communicable diseases, 53.2 percent from non-communicable diseases, three percent from maternal conditions, 29.1 percent from neonatal conditions (0 to 28 days of age), 3.4 percent from injuries, and 0.6 percent from nutritional illnesses [11]. Reproductive health, as well the health of infants and children, was a major concern.

In 1999, the infant mortality rate was 45 per 1,000 births [11] which was the highest rate in Europe, about two or three times the rate of other South Eastern European countries. Perinatal mortality was also high. In 2000, Pristina Hospital had a perinatal mortality rate of 44 per 1,000. This compares to a rate of 22 per 1,000 in 1988 [12]. In the same year, Slovenia had a perinatal mortality rate of 4.09 per 1,000; Croatia's rate was 9.37 per 1,000; Serbia and Montenegro's was 10.31 per 1,000; and Macedonia's was 15.82 per 1,000. The average rate of European Union countries was 6.78 per 1,000 [13].

Many factors contributed to these disturbing statistics, including poor obstetric standards, inadequate medical services, poverty, and malnutrition--as well as health conditions such as prematurity, asphyxia, congenital anomalies, respiratory diseases, and diarrhea [10]. Many women lacked knowledge regarding the appropriate treatment of diarrhea. Many mothers surveyed (54.6 percent) said they stopped breastfeeding when their infant had diarrhea [8].

Serious public-health issues faced children. Children suffered from a high rate of diarrhea and acute respiratory infection, a reflection of poor sanitation, lack of access to clean drinking water, and inadequate shelter [8]. As noted above, childhood vaccination was disrupted by the war, and was not universal, while improper nutrition was also a concern. Among children aged 5 to 59 months, a UNICEF survey reported stunted growth among 10 percent of children, and mild and moderate anaemia in 16 percent of children, while more than 50 percent of children between 6 and 12 years of age showed symptoms of iodine deficiency [14].

Non-communicable diseases such as cardiovascular, renal, and lung disease and chronic back pain and ulcer/gastritis were the most common adult health conditions. Because of the high smoking rate, the incidence of cancer and heart disease was increasing. Tobacco was a major contributor to morbidity and mortality. Infectious diseases were also problematic. The incidence of tuberculosis remained high at 60 to 70 cases per 100,000. There was a high case-fatality rate for some communicable diseases such as bacterial meningitis, haemorrhagic fever, viral meningo-encephalitis, shigellosis, and diarrheal diseases [14].

### External Pressures for Reform

While the Kosovo health system was in need of improvement, external actors shaped the type of reform measures selected, the scope of reform, and the timing of its implementation. Donors flooded Kosovo with billions of Euros of assistance, and the massive influx of resources in the immediate post-conflict period provided essential humanitarian relief and greatly assisted the process of reconstruction. Between 1999 and 2002, donors spent approximately 80 million Euros on the health sector, which represented the second-largest portion of the Kosovo Consolidated Budget [15]. (The largest portion was devoted to education.)

To ensure that donor funds were coordinated and sustainable, the WHO developed basic guidelines for health projects. The "Interim Health Policy Guidelines" were released in September 1999. These policy guidelines, known informally as the "Blue Book," included eight objectives:

1. Primary care would be strengthened with the development of family-medicine teams;

2. Specialist care would be provided through referral from primary care;

3. The size and location of facilities would be established through the identification of population catchment areas--which meant that some facilities would be closed, while services in others would be reduced;

4. No expansion of services should be undertaken to ensure sustainable financing. Public financing would be maintained, but other financing models would be studied;

5. Public provision of services would predominate;

6. Regulated private practice would be allowed;

7. An essential drugs program and a regulatory agency would be introduced; and

8. The provision of healthcare and employment within the system would be non-discriminatory [16].

The Blue Book was an important step in policy development in Kosovo, based on evidence of interventions in other post-conflict settings. It was non-binding on donors and NGOs, but established an important framework and point of reference for donor activity, guiding many donor interventions. The WHO produced a facility plan, which determined what facilities would remain open, the services provided, equipment lists, and staffing requirements.

In the summer of 2000, the WHO built on the momentum created by the Blue Book and developed a more ambitious health policy for Kosovo. WHO officials believed that a window of opportunity existed for reform of the healthcare system [2], a belief echoed by the World Bank in its health-planning document:

There is a relatively brief window of opportunity during which donors and international experts can have a significant impact on restructuring systems and reformulating policy before these systems and institutions become entrenched and resistant to change. A strong emphasis should be put on aid coordination to ensure complementarity in donor initiatives and a priority focus in view of limited implementation and policy development capacity in Kosovo. Development support should be conditioned on policy and structural changes aimed at providing efficiency incentives and ensuring the long-term sustainability of effective institutions and programs [17].

The reform plan had three main inputs. First, the WHO assessed major population-health issues based on the available health data. Second, they considered the vision of European healthcare systems, as outlined in the WHO's *Health for All Policy for the Twenty-First Century*. And third, they undertook consultations with Albanian physicians. A health-policy working group met regularly in Pristina, while WHO officials travelled throughout Kosovo to solicit the views of physicians practicing in other cities and towns.

Despite efforts to consult, interviews with local stakeholders demonstrated that they thought that UNMIK, the WHO and international donors were behind the reforms, with only moderate local input into the reform process. Many stakeholders believed that the strategy was pre-formulated and 'sold' during the working-group meetings. Some participants of this working group complained that "the policy framework was already ready, and we were brought into the final act." However, others were more sanguine: "The content was defined by internationals and the decision makers were internationals. This is not something wrong--it was positive as we did not have a brighter vision." Some stakeholders stressed that change was too rapid: the system was in chaos, insufficient data existed to make decisions about reform, and little preparation was undertaken for reform implementation. And as a result of these external pressures for reform, the majority of central-level stakeholders interviewed expressed doubt that the Kosovars working in the health system were committed to reforms.

### The Reform Measures: The Yellow Book's Plan

These consultations resulted in Kosovo's health-policy document, informally known as "The Yellow Book." The Yellow Book outlined an ambitious vision for the health system in Kosovo, [18] and its basic components are outlined below.

#### Primary Care

The Yellow Book committed to a primary care-focused health system. Family medicine teams operating in primary care centres would provide initial diagnoses and curative care, with the objective of treating 80 to 90 percent of presenting problems. The location of health clinics would be determined on the basis of population: facilities would have catchment populations of approximately 10,000 individuals. Larger communities would have more extensive primary care facilities known as 'family medicine centres,' while smaller communities would have small clinics known as 'punctas.' No expansion of public clinics was deemed necessary.

Family medicine centres would be responsible for diagnoses and curative care, including minor surgery and drug management; emergency care and stabilisation of emergency patients; maternal and child healthcare; and reproductive health services, including antenatal and post-natal care, as well as family planning and treatment of sexually transmitted diseases. Individuals would choose their family doctor, who would be responsible for coordinating specialist and tertiary-care services. Patients who bypassed the referral system would face a financial penalty. Prevention activities such as health education and immunisation would be run out of these centres, as would services such as home visits, palliative care, community rehabilitation, and community mental-health services.

#### Secondary and Tertiary Care

The Yellow Book outlined a system whereby patients would receive specialist care and hospitalisation upon referral only, except in emergencies. Specialists who were not working in family medicine would be hospital-based. Outpatient specialty care would be provided at hospitals and selected family medicine centres on referral. Six hospitals would provide secondary care, and tertiary care would be provided at one or two sites in Kosovo upon referral only.

Hospitals in Kosovo were not cost-effective, operating at 75 percent capacity with unnecessarily lengthy patient stays and cumbersome physical structures. The Yellow Book specified that hospital master plans would be written, outlining how to increase the efficiency of hospital services. The number of beds would be reduced in most hospitals. In addition, future budget allocations to hospitals would be based upon performance contracts and service agreements.

#### Public and Environmental Health

Kosovo's Institute of Public Health (IPH) consisted of one central institute with five regional offices. These institutes were not well connected with the rest of the health system, their equipment obsolete, and health-information systems not functioning. Under Kosovo's health policy, the IPH would be modernized and would concentrate on three areas: communicable disease control, health promotion, and water and food safety. It would also function as the technical arm of the Department of Health, providing it with timely and accurate information on public-health issues. The IPH would also guide and supervise public-health activities at the district and municipal levels.

#### Financing

No specific financing provisions were outlined in the Yellow Book. It contained a pledge that the Department would study various funding sources. Options included tax revenues, social insurance, voluntary contributions, private insurance, community insurance, co-payments, and a fee-for-service system, with the likely system being some form of pre-payment (through compulsory or voluntary health insurance). Co-payments would remain in place, as they were important sources of income and could support health-policy goals (such as the referral system).

#### Governance

The Yellow Book outlined the role of the Department of Health, which would later be transformed into the Ministry of Health. Under the Kosovo health guidelines, it would be responsible for policy, strategic planning, regulation and standard setting, monitoring to ensure adherence to regulations, human-resource planning, licensing, quality assurance, and budgeting. Several institutes, including the IPH and the Pristina University Hospital, would report directly to the department. In line with the European Union's principle of subsidiarity, oversight of primary care would rest with the municipality, but the Department of Health would ensure that municipalities adhered to central guidelines and standards.

### The Outcomes of Health Reform

Below, we assess progress made on various elements of the reform process.

#### Primary Care

The reorientation of the system towards primary care was ambitious, requiring the introduction of the family-medicine concept; the establishment of a strong interface between primary and secondary or tertiary levels of care; the management of the decentralisation process to ensure that this led to increased responsiveness to local needs rather than a deterioration in the quality of health services provided; and careful oversight by authorities to ensure that physicians did not abuse their ability to work in both the public and private sectors.

Progress has been mixed. The concept of family medicine became part of the health-system lexicon. The Kosovo Health Law enshrined family medicine as the "essential form for provision of overall health care services at the primary care level for individuals and their families" [19]. Training programs for both physicians and nurses were initiated. This training included management of Kosovo's health priorities: maternal and child health; prevention of heart and lung disease; tuberculosis; mental health; quality of care; and patient prescriptions [20]. The Ministry of Health established the main Centre for the Development of Family Medicine in Pristina in September 2002, along with eight regional Centres for Family Medicine Training. Family medicine was introduced into the curriculum of undergraduate medicine.

Despite these advances, the family-medicine system was slow to become established. Many physicians interviewed indicated that family medicine had either been "tolerated" or "resented"; only five out of 23 who responded to this question indicated that it had been received "enthusiastically." The gate-keeping role of primary care remains underdeveloped. As one doctor complained, "There is no continuity of patient follow-up, patients come and get the referral from the family medicine doctor and just go to the specialist." Family medicine faced resistance from specialists, who believed that they were in competition with family doctors. One stakeholder stated, "Non-family medicine specialists oppose the health strategy as it is based on family medicine. This is due to a conflict of interest--less patients for specialists." These specialists often redirect those arriving at hospitals to their private clinics.

Stakeholders interviewed believed the family medicine program should have been implemented more slowly and carefully. Members of family-medicine teams complained that although they received training, once back in health clinics, they returned to their old methods of work. Regional stakeholders argued that family-medicine teams did not function in their areas of responsibility.

Efforts to ensure that physicians did not abuse their ability to practice in both the public and private sectors also proved difficult. The average salary of doctors was extremely low, with primary care doctors earning only 200 Euros per month. This created an incentive to go into private practice, where doctors could earn many times that amount. The Ministry of Health lacked the regulatory capacity to oversee these private clinics. Stakeholders indicated that the quality of healthcare in the private sector was of serious concern because regulations were not respected.

#### Secondary and Tertiary Care

Reform to the secondary and tertiary levels of the health system received significantly less attention and financial support than primary healthcare reforms. One specialist complained, "there is not enough information about the future of the secondary and tertiary levels of care. No strategic plan has been created to determine how reform should progress."

Moreover, stakeholders believed that the Kosovar public still perceived primary care as a stopping point on the road to specialist care, not as a place to receive treatment. As a result, the specialist and tertiary levels remained significantly oversubscribed. Despite the population's continued reliance on hospitals, and the dysfunctional referral system, the health-sector budget in Kosovo was evenly split between primary and secondary care services, even though secondary and tertiary care were much more expensive [21]. This left hospitals under-funded for their level of activity, with few resources to maintain hospital infrastructure. While hospital master plans were developed, the funding to implement these plans was consistently lacking.

#### Public Health

Public health was made a municipal responsibility, and municipal public-health inspectors were hired. Responsibility for immunisation was transferred to primary care facilities. A health information system was put in place, but concerns remained regarding the ability of the IPH to provide reliable information to the Ministry of Health, and evidence-based policy advice.

#### Financing

The health system continued to be funded out of the Kosovo Consolidated Budget. As taxation generated more revenue over time, the amount of money allocated to the health system gradually increased. The health system received the equivalent of 41.53 million Euro in 2000, 48.5 million Euro in 2001, 40.8 million Euro in 2002, and 44.4 million Euro in 2003 [10]. These amounts remained inadequate, and the low financial capacity of the Kosovo government undermined the sustainability of the reform process. According to the Ministry of Health, in 2005 Kosovo spent 6.4 percent of its GDP on health, with 2.4 percent from public resources (the Kosovo budget); 0.7 percent from donor resources, and 3.2 percent from private sources. Private expenditure through out-of-pocket expenses for private services and pharmaceuticals, co-payments, and under-the-table payments was higher than public expenditure. Total public-health expenditure was about 22 Euro per capita--in Croatia it is about 320 Euro per capita [10].

These additional costs for individuals attempting to access the health system created barriers to healthcare. This inability to access care when needed undermined the equity of the system. While the majority of stakeholders interviewed stated that the reforms provided better access to healthcare for rural populations and women, they argued that the reforms had resulted in less access for poorer populations.

Significant challenges faced UNMIK in reforming healthcare financing. Kosovo was poor, and providing effective healthcare in the face of resource constraints was an immense challenge. The lack of basic accounting practices also impeded progress. Budgeting systems were not sophisticated enough to hold institutions accountable. Until the summer of 2001, accounts with the Department of Health were done on Excel spreadsheets, which allowed for significant corruption. For example, the pharmaceutical budget was a single block allocation without separate allocations for hospitals, municipalities, and clinical services, and there was no coding structure for goods and services throughout the health sector.

The World Bank funded a project designed to assess the most appropriate financing system and implement the basis for that system. The Bank greatly overestimated Kosovo's governance capacity, specifically the capacity of the remnants of the Kosovo Health Insurance Fund (HIF). In 2000, the World Bank stated: "The top management is highly experienced, qualified, and motivated to resurrect Fund activities. We believe that the human capacity of the HIF could be easily and quickly mobilized if it were necessary" [17]. While this analysis formed the basis for their decision to reinvigorate the social insurance system, the HIF lacked the capacity to undertake basic administrative functions. Its building was heavily damaged during the war, and its Serb staff had fled, while the Albanian staff who returned to HIF had been out of the system for 10 years.

To build this capacity, the Health Care Commissioning Agency (HCCA) was developed as a forerunner to an insurance fund. The HCCA would initially exist within the Ministry of Health, with plans to make it an independent entity in the future. The HCCA would establish the basis for the contracting of services, necessary to split the purchaser and provider functions, with the goal of signing performance contracts with municipalities for primary care, and with hospitals for secondary and tertiary care. The HCCA would essentially buy the services that these institutions provided, stipulating the type and quality of service.

Progress in establishing the HCCA was hampered by the absence of key inputs such as accurate data, information and management systems, and reward systems. The HCCA was also charged with the task of identifying the basket of health services that would be provided free of charge. This task was undermined by the lack of data on morbidity and mortality and the lack of basic financial data. A health-insurance law has been prepared, but as of 2009, had not been passed.

#### Governance

By the summer of 2001, UNMIK faced three main tasks in the field of health. First, it administered the health system. Second, it built the foundation for a future Ministry of Health which required building managerial and technical capacity within the Department of Health, establishing a regulatory framework for the future Ministry of Health, developing a health-financing strategy, establishing human-resource policies, exercising quality control, oversight of the pharmaceutical sector, and regulating the quickly growing private sector. And third, it implemented the health-reform program.

After the central elections in November 2001, the Provisional Institutions of Self-Government (PISG) were established and the Ministry of Health was put in place. The Ministry of Health had the mandate to monitor the health situation and implement appropriate measures to prevent and control healthcare problems, develop policies and implement legislation, coordinate activities in the health sector including the management of healthcare infrastructure, develop and implement norms and standards, and oversee adherence to such standards. It was staffed by civil servants and led by an official appointed by the Prime Minister. Internationals were transformed from positions of authority within the Ministry to advisory roles.

In the first year of its formal existence (2002), the Ministry was wracked by political disputes. The first Minister was dismissed, as he reportedly did not fully respect the Ministry's hiring procedures and had made political appointments to the civil service. His cooperation with donors was minimal and sometimes hostile, and he obstructed some key developments such as the appointment of the Permanent Secretary--the highest civil servant within the Ministry of Health. The dismissal of the Minister invoked a political crisis, which further disrupted the already slow progress in fully establishing the Ministry.

Partly as a result of these disruptions, there was little activity in the Ministry of Health on implementing the Yellow Book program for reform. Apart from ongoing donor initiatives such as training of family-medicine physicians and the establishment of a health-insurance system, little attention was paid to the Yellow Book. The Ministry was preoccupied with keeping itself afloat amidst scandal and a lack of leadership.

Results were also disappointing at the local level. In some municipalities with strong political leadership and less contentious political environments, decentralisation did not result in a deterioration of primary care services. In other areas, where the capacity of municipal councils was weak, critics argued that decentralisation led to heightened corruption and reduced access to healthcare, particularly for minority communities. The majority of stakeholders interviewed believed that the decentralisation of primary care services had either made no change or had worsened the delivery of care. One stakeholder stated, "Municipalities do not have the capacity to take on these responsibilities. The centre does not have the capacity to monitor municipalities and they are left to themselves." Some stakeholders believed that responsibilities should have been transferred gradually, when municipalities developed management capabilities.

#### Government Capacity to Implement Reforms

The Ministry of Health had little time or human resources to develop an implementation plan for health reform. Under the UNMIK's Department of Health, regulations were in place (although the Department had little capacity to enforce them), a payroll established, procurement of medicines and supplies undertaken, and rudimentary oversight of local institutions provided. Although the Department was successful in putting in place a basic administrative structure and a rudimentary regulatory framework, it did not have the capacity to plan for or undertake reforms. No one within the UN Department had experience working in a Ministry of Health, donors did not provide the Department with the necessary support, and the Department was woefully short-staffed. The staff who were in place were preoccupied with the basic tasks of administering the healthcare system, coordinating donor/NGO activity, and beginning the gradual process of transferring responsibility for healthcare functions to municipalities.

The civil service was not fully established until after central elections were held in 2001, which was a missed opportunity to begin the process of building an independent public service prior to the election of elected officials. Moreover, civil-service salaries were extremely low, and government departments lacked the ability to compete with international agencies for staff.

There was no official, sector-wide strategy beyond the ambitious goals of the Yellow Book. The Ministry of Health did not communicate its vision for healthcare. The majority of stakeholders indicated that discussion of the reforms with Kosovo health professionals was moderate or infrequent. They expressed concern with the lack of discussion surrounding reforms--particularly after the initial consultations that the WHO had undertaken after the Yellow Book was formulated. While the majority of stakeholders also stated that the reforms were not sufficiently communicated to the public, some noted that extensive public communication was not possible at the time.

The majority of stakeholders interviewed believed that the Ministry did not act sufficiently to implement reforms. This view was particularly marked among central-level stakeholders. As one stakeholder stated, "The Ministry did not have the capacity or will to implement the policy. They designed regulations as they needed, but they did not have any systematic plan in place to promote health policy. The right people were not in the right places." Stakeholders did not believe that the services available at primary healthcare facilities met the objectives of the reform program, and the vast majority of stakeholders agreed that the Ministry of Health was not able to enforce its standards in private healthcare clinics.

Government capacity was not enhanced by the activities of donors. Donors had short time horizons and dispersed most of their programming funds in the first two years of the mission (1999-2001). While this ensured that immediate humanitarian needs were met, it undermined efforts to achieve longer-term development goals. Short time horizons made donors risk-averse, as they had to achieve certain objectives within a limited period of time. Donors often had specific national objectives for their money, including support to national non-governmental organisations and specific national projects ('planting their flag'). They focused on quantitative outputs, such as the number of health clinics re-equipped, and nurses trained. Projects that would contribute to the broader reform process such as establishing standardized training and building the capacity of the Kosovo civil service were secondary considerations. While donors coordinated their activities, they did not engage in a sector-wide approach. Most donor funds went to hundreds of NGOs, not the Department of Health, and donors did not report to the Department. Coordination and collaboration was strictly voluntary.

The contentious nature of politics in the immediate post-conflict period also undermined Kosovo's administrative capacity. This capacity was already weak due to the consequences of the disruption of government during the 1990s, the inexperience of Kosovo's politicians, the sluggish rate of the UN's establishment of government administration, and Kosovo's economic weakness. The ongoing struggles between Albanian political parties, Albanians and minorities groups undermined the ability of the Ministry of Health to implement the health reform agenda.

#### Health Reform Outcomes

Table 1 (see appendix) outlines the objectives of health reform as presented in the Yellow Book, and summarizes progress made towards meeting these objectives. As evident in this table, family doctors have been trained, responsibility for primary care has been transferred to the municipal level, immunisation coverage has increased, and some maternal and child health indicators have improved. Yet many key reform initiatives, such as building the strength of primary care and establishing an effective health-financing system, were not fully implemented.

**Table 1:** Progress in Meeting Health-Reform Goals

	Reform Objectives	Reform Progress
**Primary Care**	Location and services offered by family-medicine centres would be based on population. Family doctors would have patient lists, and be responsible for diagnoses and curative care, reproductive, maternal and child health, and emergency care and stabilisation. Family doctors would be responsible for coordinating specialist and tertiary-care services. Private practice would be allowed, and physicians would be allowed to practise in both the public and private sectors, but institutions must be approved and regulated.	The WHO established a facility master plan based on capitation, which guided rehabilitation and staffing. In minority areas, some facilities were opened that were not included on the master plan. Family-medicine training established. Serious impediments exist: patient registration is not universal, gate-keeping role of primary care underdeveloped, and specialists resist primary care role. Ministry lacks the capacity to regulate the private sector, and there are accounts of physicians redirecting patients from the public sector to their private clinics.

**Secondary and Tertiary Care**	Patients would receive specialist care and hospitalisation upon referral only, except in emergencies. Hospital Master Plans will establish a vision for increasing the efficiency of hospitals.	Patients often bypass the primary care level to receive direct treatment by specialists. Hospitals were overburdened and under-resourced. While Hospital Master Plans were developed, the Ministry lacked the resources to implement these plans.

**Public Health**	The Institute of Public Health would focus on communicable disease control, health promotion, and water safety. The institute would operate as the technical arm of the Department of Health, providing it with information on public-health issues.	Oversight of public health transferred to municipalities, public-health inspectors operate at the municipal level. Immunisation transferred to primary care. Health information system established, but the ability of the Institute of Public Health to provide timely and accurate analysis to the Ministry of Health questioned.

**Healthcare Financing**	No commitment was made to any financing system, but a pledge was made to study the merits of various alternatives. Some form of pre-payment system would be established through compulsory or voluntary insurance. Co-payments would be maintained.	Equity marred by significant private expenditures (including under-the-table payments) System funded out of the Kosovo Consolidated Budget. Precursor to a social-insurance system, the Health Care Commissioning Agency (HCCA), established. Establishment of the HCCA and performance-based contracting has been undermined by the absence of accurate data, and information and management systems. The failure to establish a transparent accounting system prior to the HCCA slowed efforts to implement health-financing.

**Organisation and Governance**	The Ministry of Health would be responsible for policy, strategic planning, and regulation and standard setting. Responsibility for primary care would be decentralized to municipal level.	The Ministry initially undermined by political turmoil, including changes of Minister and controversy surrounding the appointment of senior civil servants. Turmoil undermined its capacity to implement reforms. Oversight for primary care became the responsibility of the municipalities in 2001. Municipalities slow to establish oversight structures, and capacity of municipalities varies.

## What Does this Mean for Post-Conflict Reform?

Health reform is a complex undertaking, and it can take years of resources and effort to produce meaningful change. Yet trends in health reform can be evaluated, and the Kosovo case study sends a cautionary note to those planning ambitious reforms in post-conflict settings.

### What Went Right

Important lessons from other post-conflict contexts were applied in the case of Kosovo. The WHO assumed a coordination function and established a strategic-planning document to guide investments in the health sector. The WHO formulated basic health guidelines soon after the conflict ended. Donor funds were then used to build the foundation for health reform. A facility master plan guided the rehabilitation of health facilities. Weekly coordination meetings were held. These important developments took place in a difficult context with a multiplicity of donors and NGOs and a weak government in the form of UNMIK.

Moreover, Kosovo's health policy provided stakeholders in the health sector - donors, international agencies, non-governmental organisations, and Kosovo health professionals - with an opportunity to outline a shared vision for the health sector. Despite the pessimism generally expressed by stakeholders, all hoped that in 15 years the health system would reflect the vision outlined in the health-policy document. The health policy document guided donor responses and ensured a degree of coherence in rehabilitation and reform efforts. Training of family doctors took place, family-medicine centres were established, and the HCCA acted as the precursor to a health-insurance fund. Legislation devolved responsibility for primary care and public health to the local level.

### What Went Wrong: Confirming the Hypotheses

Although much was accomplished, reforms largely failed to meet their objectives. Below, we address each hypothesis and summarize the research that affirms these hypotheses.

*H1: External actors drove the health reform process: the policies selected reflected the objectives of the international community*.

The reform program was clearly driven by the World Health Organization and donors such as the Word Bank. While an effort was made to consult with Albanian health professionals, stakeholders believed that they were being 'sold' the health care program, rather than having input into the design of the reform measures.

*H2: Donors believed that reform could be achieved in a compressed time period, and gave more priority to the design than the implementation of reforms*.

Donors and the WHO argued that an important window of opportunity existed for reform: stakeholders were not in a position to block reform, and donor resources were relatively plentiful. However, the capacity needed for this type of radical change was seriously underestimated. Stakeholders noted that "The health system changed too quickly from one system to another, and such dramatic change was impossible with all the post-war problems."

The problems posed by the compressed time frame for reform were exacerbated by the failure to undertake effective policy planning. While the policy document (the Yellow Book) was developed in consultation with donors and health professionals, no similar process took place to develop an implementation plan. One stakeholder complained that, "there were no preparations for implementation, no assessment of financial, human or management resources." As a result, there was little reflection about the possible impediments to reform, and the necessary steps to achieving reform objectives. Health reform cannot be effectively built on a weak foundation.

H3: State capacity in the post-conflict period is low, and external actors do not recognize the importance of state capacity in health reform

As outlined above, the United Nations Department of Health, and later the Kosovo Ministry of Health, was expected to undertake three objectives: first, coordinate donors and NGOs during the rehabilitation program; second, oversee the administration of the health system; and third, implement an ambitious program of reform. The Department was expected to meet these three goals with few resources. It was initially short-staffed and completely overstretched. While international staff provided competent technical advice on public-health issues, they lacked experience working within government and could not guide the transformation of the Department into a government ministry. Because of these weaknesses, the Department lacked the capacity to exercise a strong planning role. There was no sector-wide planning approach, and no implementation plan for the Yellow Book was developed.

Capacity was further undermined by the post-conflict political context. The health system was an important arena for political struggle. The KLA appointed heads of hospitals and primary clinics immediately after the war, but many of these appointments were changed after the rival political party - the Democratic League of Kosovo (LDK) won elections at the municipal level. After the central elections in 2001, the new Minister of Health introduced political appointments in the Ministry and throughout the health system, which resulted in his dismissal. This politicisation distracted Kosovo officials from the reform program and impeded progress towards meeting reform goals. When the Department of Health was transformed into a Ministry, political problems undermined the transition process. The first Minister of Health was dismissed for incompetence, and the most senior civil-servant post in the Ministry--the Permanent Secretary--remained unfilled for many months. This political instability also contributed to slowing down the implementation of reforms.

**Figure 2:**
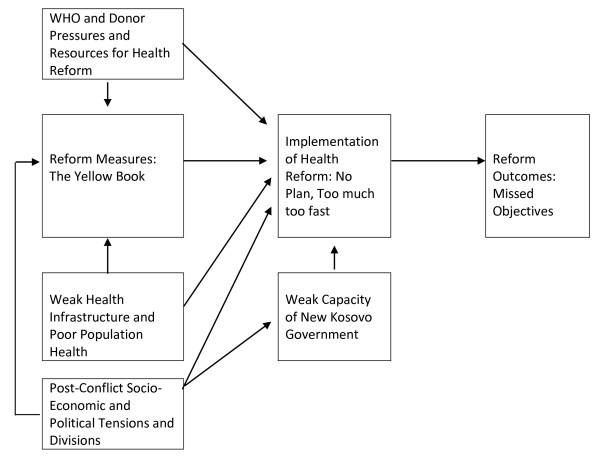
**Application of the Conceptual Framework to Kosovo Case**.

Figure 2 demonstrates how these factors undermined progress in reforms, and applies the framework for analyzing post-conflict health reform to the Kosovo case study.

## Conclusion: Implications from the Case Study

This case study has both research and policy implications. It underscores the need for further study of health reform efforts in post-conflict areas. Such case studies could determine the role of external pressure for reform, how such pressure influenced the time frame for reform, and how the political environment affected the implementation of health reforms. Such studies could also assess the success of different types of reform measures - particularly financing reforms - to establish an evidence base for the most effective health interventions and to ensure that such interventions enhance rather than detract from efforts to re-establish peace in conflict-affected societies. While dramatic health reform measures, such as those attempted in Kosovo take years to implement, progress has not been promising, and the Kosovo reform process should serve as a warning for those hopeful for dramatic social change in post-conflict periods.

This case study also has several policy implications, although more research that corroborates these findings is needed to strengthen the evidence base. First, while health reform measures should reflect evidence, domestic stakeholders know the implementing environment better than representatives from donor and multilateral agencies. Health reform measures must be revised to reflect the concerns and reservations of stakeholders. Second, although rebuilding the health system should be guided by a clear plan, more attention needs to be paid to ensuring that sufficient resources, time, and capacity exist to implement the plan. The focus should be on the building blocks of a health system such as health financing and information systems, and ensuring that the timeframe for health reform is realistic given the social, political and economic context. And third, the state is an integral component of any health system. Post-conflict reconstruction efforts need to either contribute to building state capacity or incorporate weak state capacity into the design of health reform measures.

## Abbreviations

KLA: Kosovo Liberation Army; HCCA: Health Care Commissioning Agency (Kosovo); HIF: Health Insurance Fund (Kosovo) LDK: Democratic League of Kosovo; NATO: North Atlantic Treaty Organisation; PISG: Provisional Institutions of Self-Government (Kosovo); UNICEF: United Nations Children's Fund; UNMIK: United Nations Interim Administrative Mission in Kosovo; WHO: World Health Organisation.

## Competing interests

VP worked for the Canadian International Development Agency as a Health Advisor from March 2000 to September 2000, directly engaging in the reform process. She also undertook a professional attachment for her DrPH at UNMIK's Department of Health from June 2001-October 2001.

## Authors' contributions

VP conceived of the study, undertook the research design and oversaw the interviews with the stakeholders.  ES supervised the research.  Both authors approved the final manuscript.
